# Supplementary material for: DMRforPairs: identifying Differentially Methylated Regions between unique samples using array based methylation profiles
Source: BMC Bioinformatics. 2014 May 15;15:141. doi: 10.1186/1471-2105-15-141 (PMC4046028; doi:10.1186/1471-2105-15-141)
Supplement: Additional file 2 — DMRforPairs output for the comparison of A431-MCF7 and NA17018-NA17105. Please start from the HTML files in each folder. Available via the BMC Bioinformatics website. [file 1471-2105-15-141-S2.zip › 1394847754114233_MOESM2_ESM/A431_MCF7/figures/20619.pdf]

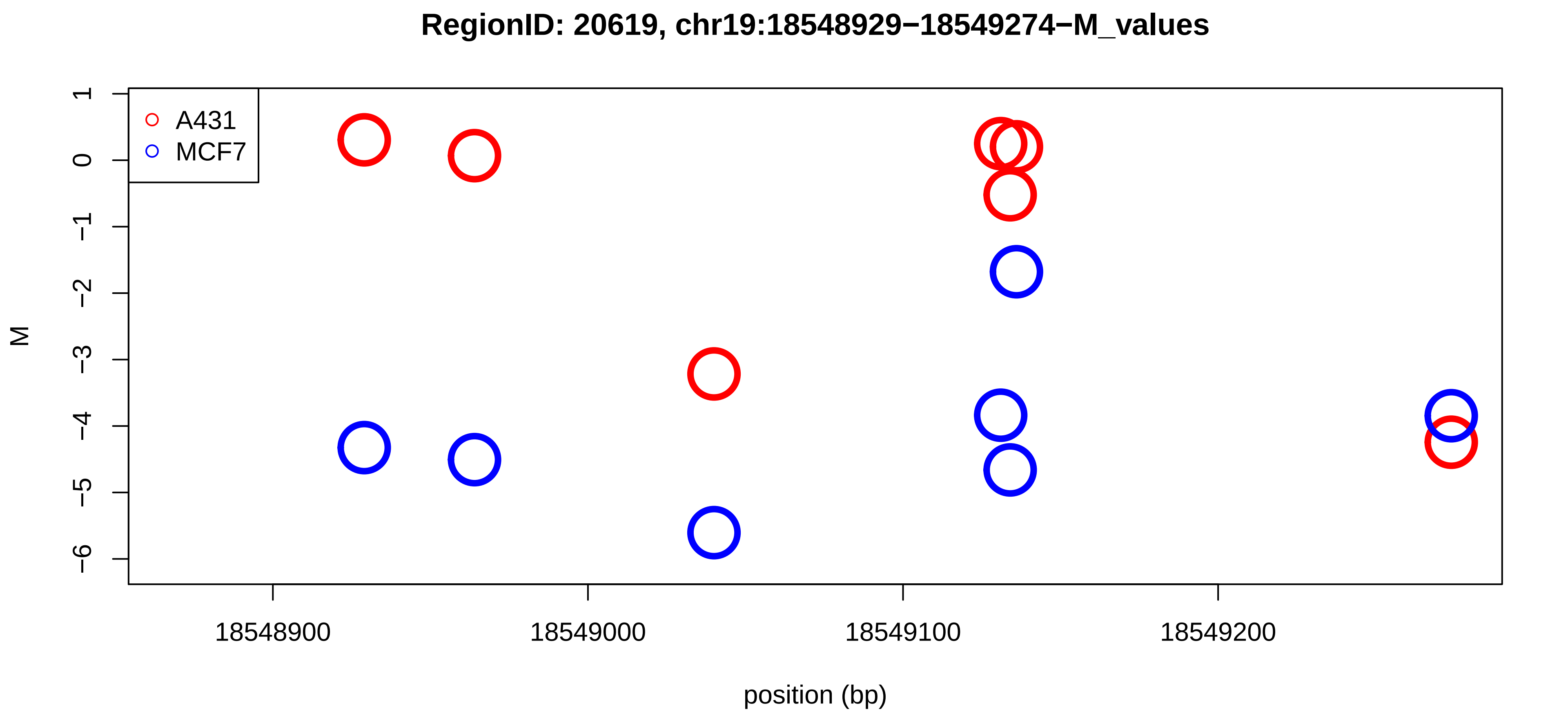
RegionID: 20619, chr19:18548929−18549274−M_values
A431
MCF7
M
1
0
−1
−2
−3
−4
−5
−6
18548900
18549000
18549100
18549200
position (bp)

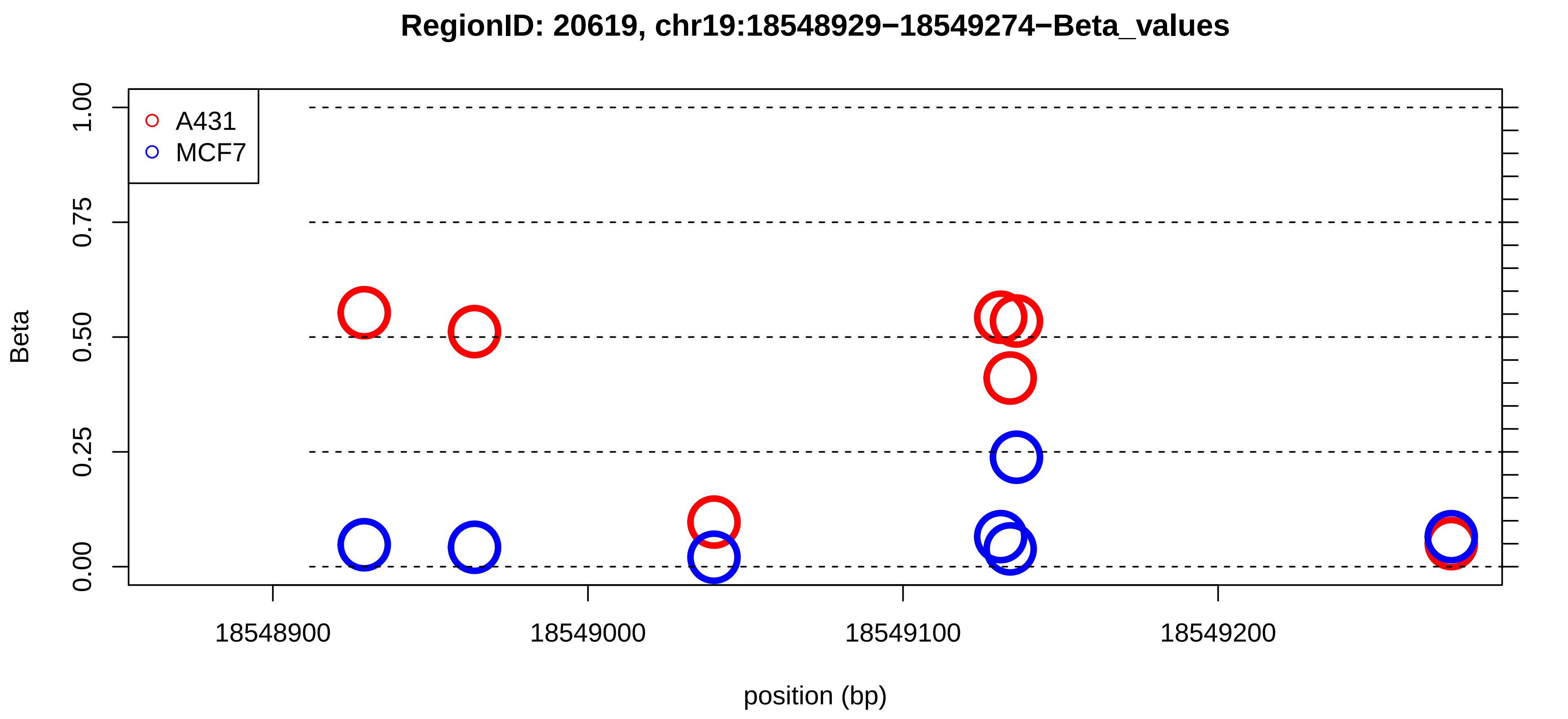
RegionID: 20619, chr19:18548929−18549274−Beta_values
A431
MCF7
Beta
1.00
0.75
0.50
0.25
0.00
18548900
18549000
18549100
18549200
position (bp)

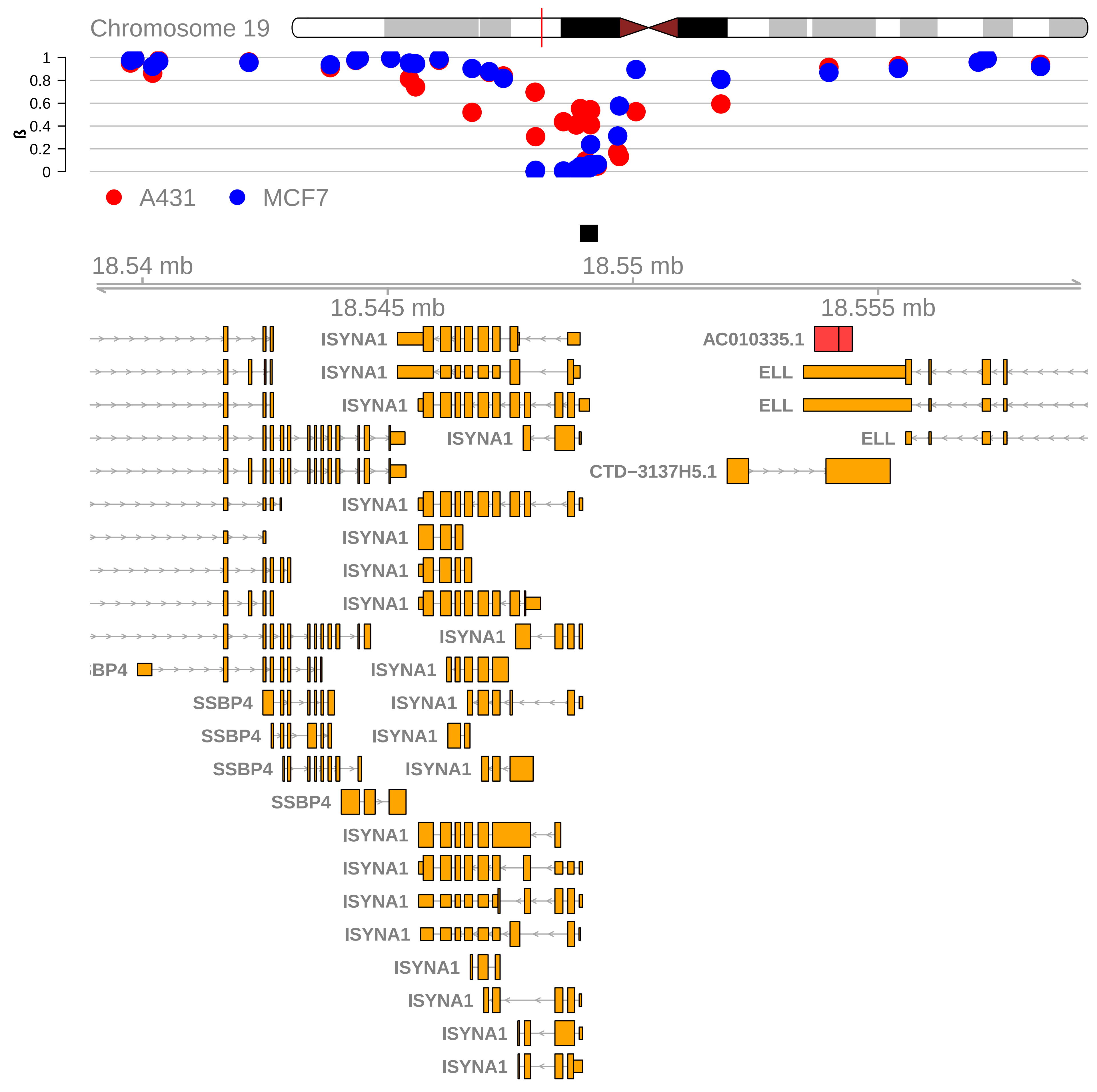

Chromosome 19
β
1
0.8
0.6
0.4
0.2
0
A431
MCF7
18.54 mb
18.55 mb
18.545 mb
18.555 mb
ISYNA1
AC010335.1
ELL
CTD−3137H5.1
SSBP4
